# Assessment of the Anti-Allodynic and Anti-Hyperalgesic Efficacy of a Glycine Transporter 2 Inhibitor Relative to Pregabalin, Duloxetine and Indomethacin in a Rat Model of Cisplatin-Induced Peripheral Neuropathy

**DOI:** 10.3390/biom11070940

**Published:** 2021-06-24

**Authors:** Andy Kuo, Laura Corradini, Janet R. Nicholson, Maree T. Smith

**Affiliations:** 1 Centre for Integrated Preclinical Drug Development, Faculty of Medicine, School of Biomedical Sciences, The University of Queensland, Brisbane, QLD 4072, Australia; a.kuo1@uq.edu.au; 2 Boehringer Ingelheim Pharma GmbH and Co. KG, 88400 Biberach, Germany; laura.corradini@boehringer-ingelheim.com (L.C.); janet.nicholson@boehringer-ingelheim.com (J.R.N.)

**Keywords:** glycine transporter, glycine receptor, analgesics, peripheral pain, cisplatin, chemotherapy, rat, pregabalin, indomethacin, duloxetine

## Abstract

Cisplatin, which is a chemotherapy drug listed on the World Health Organisation’s List of Essential Medicines, commonly induces dose-limiting side effects including chemotherapy-induced peripheral neuropathy (CIPN) that has a major negative impact on quality of life in cancer survivors. Although adjuvant drugs including anticonvulsants and antidepressants are used for the relief of CIPN, analgesia is often unsatisfactory. Herein, we used a rat model of CIPN (cisplatin) to assess the effect of a glycine transporter 2 (GlyT2) inhibitor, relative to pregabalin, duloxetine, indomethacin and vehicle. Male Sprague-Dawley rats with cisplatin-induced mechanical allodynia and mechanical hyperalgesia in the bilateral hindpaws received oral bolus doses of the GlyT2 inhibitor (3–30 mg/kg), pregabalin (3–100 mg/kg), duloxetine (3–100 mg/kg), indomethacin (1–10 mg/kg) or vehicle. The GlyT2 inhibitor alleviated both mechanical allodynia and hyperalgesia in the bilateral hindpaws at a dose of 10 mg/kg, but not at higher or lower doses. Pregabalin and indomethacin induced dose-dependent relief of mechanical allodynia but duloxetine lacked efficacy. Pregabalin and duloxetine alleviated mechanical hyperalgesia in the bilateral hindpaws while indomethacin lacked efficacy. The mechanism underpinning pain relief induced by the GlyT2 inhibitor at 10 mg/kg is likely due to increased glycinergic inhibition in the lumbar spinal cord, although the bell-shaped dose–response curve warrants further translational considerations.

## 1. Introduction

According to the World Health Organization (WHO), cancer is the second leading cause of death globally and accounts for about one in six cases involving death [1], with approximately 70% of these occurring in low-income and middle-income countries [2,3,4].

Cisplatin is a platinum-based cancer chemotherapeutic agent included in the 20th WHO Model List of Essential Medicines that include the most important medications in a basic healthcare system [5]. Cisplatin has been in widespread use for more than four decades for the treatment of many commonly occurring solid tumor types including colorectal cancer, lung, ovarian, bladder, breast, prostate, melanoma and testicular cancers [6,7,8]. Cisplatin is generally considered the most toxic amongst platinum-based drugs [6,7]. Peripheral neuropathy prevalence rates for platinum-based cancer chemotherapy agents, such as cisplatin, occurs in about 30% of patients [9,10,11,12]. Despite their toxicity, platinum-based cancer chemotherapeutic agents, including cisplatin, are commonly used in limited resource settings for their low cost, ease of administration and good efficacy for treating various solid tumor types.

Chemotherapy-induced peripheral neuropathy (CIPN) is a major dose-limiting side effect of several first-line chemotherapeutic agents, including cisplatin. Given the high prevalence of common cancers treated with chemotherapeutic agents, CIPN affects several million patients annually worldwide. The number of cancer survivors is expected to increase by 35% from 13.7 million in 2012 to 18 million people by 2022 [13,14].

CIPN is a type of peripheral neuropathic pain that is difficult to alleviate adequately. Patients may report sensory abnormalities including alterations in sensory perception, tingling, numbness, burning, increased mechanical, cold or heat sensitivity in the feet and/or hands in a stocking and glove distribution [7,15]. An important aspect of platinum-based CIPN is the “coasting” phenomenon, whereby peripheral neuropathic pain may worsen for several months following the discontinuation of chemotherapy [9]. Cisplatin-induced neurotoxicity is irreversible in more than 50% of patients once it is established [11,16,17]. CIPN severely impairs the patient’s quality of life (QOL) and it may result in dose reduction or even treatment cessation in extreme cases [18,19]. Due to the high prevalence of CIPN and its negative impact on the QOL of patients with cancer as well as cancer survivors, CIPN constitutes a major problem not only for the patients themselves but also for their caregivers and health care providers. The total societal cost of CIPN was estimated to be USD 4908 per episode [20] or USD 17,344 higher per patient with CIPN compared with patients not experiencing CIPN [21].

Currently, there are no effective treatments for alleviating CIPN. Clinical practice guidelines promulgated by the American Society of Clinical Oncology (ASCO) do not recommend any agent for the prevention of CIPN [15,22]. In the ASCO guidelines for the treatment of established CIPN, a moderate recommendation was made for the antidepressant, duloxetine. However, this recommendation was based on modest benefit that is much less than desirable [23]. Clinical use of opioid analgesics does not adequately treat CIPN [24,25] and these agents are often associated with dose-limiting side effects [24]. Given the current limited treatment options for CIPN, efficacious and well-tolerated agents for alleviating this major unmet clinical need are required.

The glycine transporters GlyT, type 1 (GlyT1) and type 2 (GlyT2) are Na^+^/CL^−^ dependent neurotransmitter transporters responsible for L-glycine reuptake into central nervous system neurons and astrocytes [26]. These two transporters have differential expression patterns such that GlyT1 is predominantly expressed in the neocortex, thalamus and hippocampus, while GlyT2 is predominantly expressed in the brainstem, dorsal horn of the spinal cord (laminae II and III) and the cerebellum [26,27]. Hence, inhibition of GlyT1 modulates glutamatergic neurotransmission through NMDA receptors, while inhibition of GlyT2 modulates glycinergic and GABAergic pathways [26]. Thus, selective GlyT2 inhibitors hold promise as novel analgesics that could facilitate inhibitory glycinergic neurotransmission in the dorsal horn of the spinal cord without activation of excitatory NMDA receptors [28]. By inhibiting GlyT2, synaptic glycine concentrations will increase in the dorsal horn of the spinal cord with the potential to increase inhibitory signaling and decrease pro-nociceptive signaling and, thus, induce pain relief [27].

The glycine transporter 2 (GlyT2) inhibitors, ORG25543 and ALX1393 are irreversible and reversible inhibitors, respectively [26,28]. These agents have been shown to alleviate mechanical and cold hyperalgesia in the chronic constriction injury rat model of neuropathic pain and the formalin-induced nociceptive pain model [26,28]. ORG25543, however, was cardiotoxic as it had potent hERG inhibitory activity and it lacked oral bioavailability [29,30]. By comparison, ALX1393 had poor blood–brain barrier penetration and, interestingly, it also had activity on GlyT1 [31,32].

Herein, we used an optimized rat model of cisplatin-induced CIPN to assess the efficacy of a GlyT2 inhibitor tool compound relative to representative agents of several other pharmacological classes for the relief of mechanical allodynia and mechanical hyperalgesia in the bilateral hindpaws. This GlyT2 inhibitor is a 3-pyridyl amide derivative of ORG25543 (Supplementary Figure S1) that has less hERG inhibition, good GlyT2 selectivity, good potency, good oral bioavailability and a suitable pharmacokinetic profile [29,30].

## 2. Materials and Methods

### 2.1. Experimental Animals

This study was conducted in accordance with the guidelines set out in the Australian Code of Practice for the Care and Use of Animals for Scientific Purposes, 8th edition, [33]. Animal ethics approval was obtained from the University of Queensland Animal Ethics Committee prior to initiation of the study. Male Sprague-Dawley rats were purchased from the Animal Resources Centre (Perth, WA, Australia).

One hundred and five male Sprague-Dawley (SD) rats (Animal Resources Centre, Canning Vale, Australia) weighing from 180 to 200 g (6 to 8 weeks of age) upon arrival were housed in a purpose-built Physical Containment Level 2 (PC2) animal holding facility. Animals were housed in groups of two or three in individually ventilated cages (BioZone Global, Thornehill, UK) in a temperature-controlled facility (23 (±3) °C; mean ±SD) equipped with a 12 h/12 h light/dark cycle. Rodent chow (Specialty Feeds, Glen Forrest, Australia) and tap water were freely available to rats. Animals were maintained in cages with recycled paper bedding material (FibreCycle Pty Ltd., Yatala, Australia). Kimwipes (Kimberly-Clark Professional, Milsons Point, Australia), rodent hutches (Able Scientific, Welshpool, Australia) and Rat Chewsticks (Able Scientific, Welshpool, Australia) were available in the cages as environmental enrichments. Rats had access to food (Specialty Feeds, Glen Forrest, Western Australia, Australia) and tap water ad libitum. Rats were acclimatized for at least 4 days before commencing any experimentation.

### 2.2. Drugs and Reagents

Pregabalin was purchased from Toronto Research Chemicals (North York, ON, Canada). Duloxetine, indomethacin, the glycine transporter 2 (GlyT2) inhibitor (N-(6-((1,3-dihydroxypropan-2-yl)amino)-2-(dimethylamino)pyridin-3-yl)-3,5-dimethoxy-4-(4-(trifluoromethyl)phenoxy) benzamide) and Natrosol™ hydroxyethylcellulose were provided by Boehringer Ingelheim Pharma GmbH & Co. KG. Tween 80 was purchased from Sigma Aldrich (St Louis, MO, USA).

### 2.3. Induction of Cisplatin-Induced Peripheral Neuropathy (CIPN) in Rats

The cisplatin dosing regimen used to induce peripheral neuropathy was reported previously by our group [34]. Briefly, once body weights were in the range of 200 to 240 g, rats were restrained gently and administered a subcutaneous (s.c.) injection of 2 mL of sterile saline at 5 min prior to cisplatin injection as a means to induce hyper-hydration and prevent renal toxicity [34,35]. Rats were administered four single intraperitoneal (i.p.) bolus doses of cisplatin at 3 mg/kg at once weekly intervals on days 0, 7, 14 and 21 to produce a cumulative cisplatin dose of 12 mg/kg.

### 2.4. Test Compound Administration

CIPN-rats with fully developed mechanical allodynia and mechanical hyperalgesia in the bilateral hindpaws (PWTs ≤ 6 g; PPTs ≤ 80 g respectively) were randomly assigned to receive a single oral (PO) bolus dose of pregabalin, duloxetine, indomethacin, vehicle or the GlyT2 inhibitor. Dosing solutions were prepared using 0.5% Natrosol™ hydroxyethylcellulose and 0.01% Tween 80 as the vehicle. Individual CIPN-rats received a maximum of five doses of the test compound or vehicle commencing on day 28 post-first cisplatin injection with at least 2 days of “wash-out” between consecutive doses. All behavioral tests and animal welfare assessments were carried out in the light phase between 8:00 a.m. and 6:30 p.m.

### 2.5. Animal Health Assessments

The general health of animals was assessed prior to each cisplatin injection and at intermittent intervals (weekly or fortnightly) during the study. Health assessments comprised the following: (1) body weight; (2) clinical observations; (3) blood haematocrit levels; (4) urine analysis; (5) body temperature measurements.

Body weights were measured in individual rats once daily for two days prior to the first cisplatin injection, on the day of each cisplatin dose and once daily for two days after each cisplatin injection (i.e., day −2, −1, 0, 5, 6, 7, 12, 13, 14, 19, 20, 21, 26 and 27).

Clinical observations comprised the following observations: (1) skin and fur; (2) eyes and mucus membranes; (3) respiratory and circulatory function; (4) gait and posture; (5) behavior; (6) clonic tremors or convulsions; (7) tonic tremors or convulsions. Animals were observed prior to the first cisplatin injection and then once daily for two days after each cisplatin injection (i.e., day −1, 1, 2, 8, 9, 15, 16, 22 and 23).

Blood haematocrit levels were determined prior to the first cisplatin injection and then at fortnightly intervals (i.e., day −1, 13 and 27) until the end of the study. Blood samples (~60 μL) were collected from the tail vein of individual rats placed into pre-labelled haematocrit tubes (75 mm; Drummond Scientific Co., Broomall, PA, USA). The samples were centrifuged for 10 min at room temperature using a haematocrit rotor at 10,000 rpm/11,865 g (Damon/IEC division, IEC MB centrifuge, Micro Hematocrit; Hawksley & Sons Ltd., West Sussex, UK). The haematocrit levels were determined visually using a micro-hematocrit capillary tube reader chart.

For measurement of body temperature, rats were briefly anaesthetized using isoflurane (3% delivered in 100% oxygen) to enable rectal insertion of the thermometer probe (Precision Thermometer, YSI 4600; SDR Clinical Technology, Sydney, NSW, Australia).

For the urine analyses, rats were individually housed in metabolic cages for 2 h with a polished stainless-steel funnel and collection vessel underneath the cage for urine collection. Urine indices of kidney function were determined using Reagent Strips 10SG for Urinalysis (Livingston International, Mascot, NSW, Australia). Specifically, glucose, bilirubin, ketones, blood (erythrocytes), specific gravity, pH, protein, urobilinogen, nitrite and leucocytes were measured (refer to Supplementary Information for details).

### 2.6. Assessment of Mechanical Allodynia in the Bilateral Hindpaws of CIPN-Rats

Assessment of the time course for the development of mechanical allodynia (hypersensitivity to applied non-noxious mechanical stimuli) in both hindpaws of CIPN-rats was performed using calibrated von Frey filaments (Stoelting Co, Wood Dale, IL, USA) as described previously [36]. Briefly, rats were placed in wire mesh testing cages and permitted to acclimatize to the experimental setup (room, cage and handling) for approximately 30 min prior to testing. The paw withdrawal thresholds (PWTs) for each of the left and right hindpaws were the mean of three readings for each hindpaw, with a 5 min interval between consecutive measurements. Mechanical allodynia was considered to be fully developed in the hindpaws when the mean PWT was ≤6 g. For animals administered single bolus doses of the GlyT2 inhibitor, pregabalin, duloxetine, indomethacin or vehicle, their PWTs were measured pre-dose and at the following post-dosing times (0.5, 1, 2 and 3 h).

### 2.7. Assessment of Mechanical Hyperalgesia in the Bilateral Hindpaws of CIPN-Rats

Assessment of the time course for the development of mechanical hyperalgesia (hypersensitivity to applied noxious mechanical stimuli) in both hindpaws of CIPN-rats was performed using the Randall Selitto apparatus (Ugo Basile, Gemonio, VA, Italy) as described previously [36]. Briefly, rats were allowed to acclimatize to the experimental setup (room and handling) for approximately 30 min prior to testing. The paw pressure thresholds (PPTs) for each of the left and right hindpaws were the mean of three readings for each hindpaw, with a 5 min interval between consecutive measurements. Mechanical hyperalgesia was considered as fully developed in the hindpaws when the mean PPTs ≤ 80 g. For animals administered single bolus doses of the GlyT2 inhibitor, pregabalin, duloxetine, indomethacin or vehicle, their PPTs were measured pre-dose and at the following post-dosing times (0.5, 1, 2 and 3 h).

### 2.8. Data and Statistical Analyses

Data are presented as the mean ± standard error of the mean (SEM) for the PWTs and the PPTs for the left and right hindpaws in each treatment group. Delta PWT or PPT (ΔPWT or ΔPPT respectively) values were calculated by subtracting pre-dosing PWT or PPT values from post-dosing PWT or PPT values, respectively, and any negative ΔPWT or ΔPPT values were arbitrarily assigned a value of 0. For individual CIPN rats, the extent and duration of action were determined by calculating the area under the ΔPWT or ΔPPT vs. time curve. One-way analysis of variance (ANOVA) with Dunnett’s Multiple Comparison test was performed on the mean (± SEM) ΔPWT or ΔPPT AUC values for groups of CIPN rats administered single oral bolus doses of pregabalin, indomethacin, duloxetine and the GlyT2 inhibitor relative to the animals administered single oral bolus doses of vehicle. GraphPad™ Prism version 9.0.0 was used for all data and statistical analysis. The statistical significance criterion was *p* ≤ 0.05.

## 3. Results

### 3.1. Temporal Development of Hindpaw Hypersensitivity in CIPN-Rats

**Figure 1:**
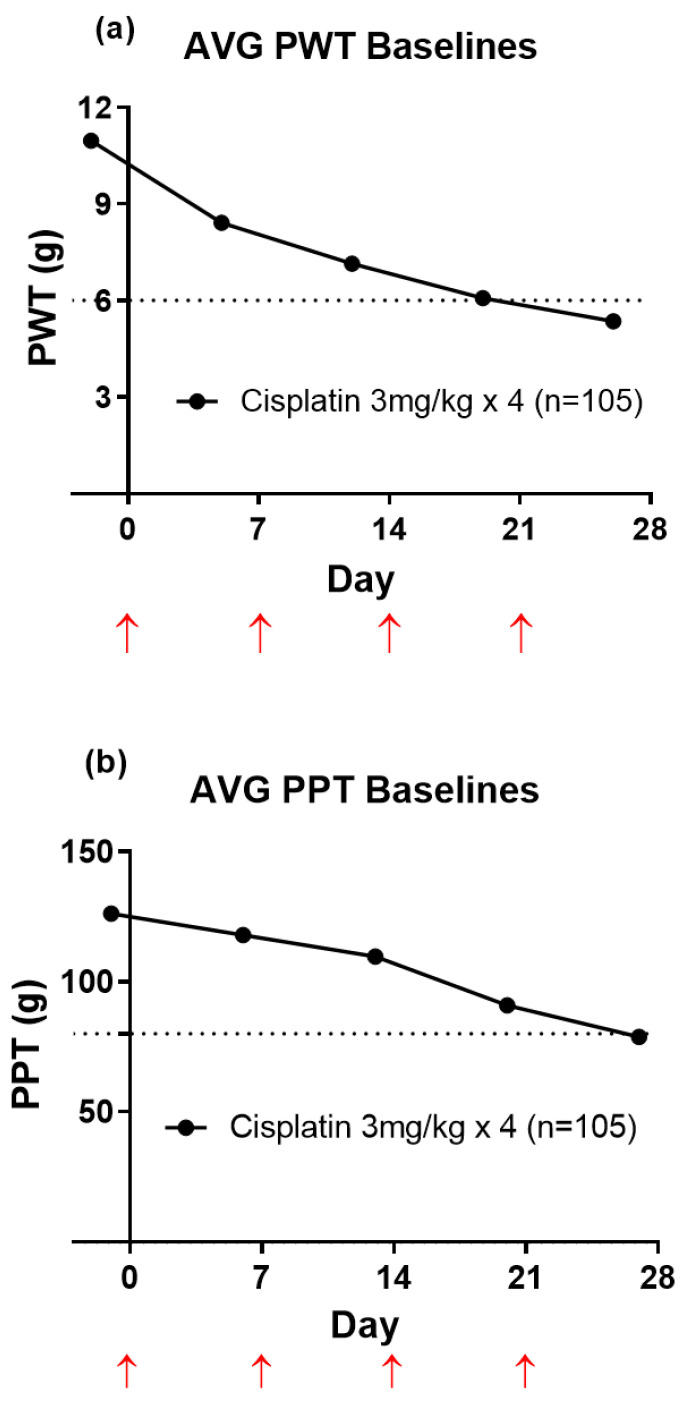
(**a**) Mean (±SEM) baseline paw withdrawal threshold (PWT) versus time curves. The horizontal dotted line in Panel (**a**) indicates fully developed mechanical allodynia in the bilateral hindpaws (PWT ≤ 6 g). (**b**) Mean (±SEM) baseline paw pressure threshold (PPT) versus time curves. The horizontal dotted line in Panel (**b**) indicates fully developed mechanical hyperalgesia in the bilateral hindpaws (PPT ≤ 80 g). Red upward pointing arrows indicate the days of each cisplatin (3 mg/kg; IP) injection. Please note that the error bars have been added, but they cannot be seen as the data is very tight (n = 105).

There was temporal development of mechanical allodynia (PWT; Figure 1a) and mechanical hyperalgesia (PPT; Figure 1b) in cisplatin-treated rats that was fully developed by four weeks after the initiation of the first cisplatin injection (3 mg/kg IP). Mechanical allodynia was fully developed when the bilateral hindpaw PWT values were ≤6 g. Mechanical hyperalgesia was fully developed in the bilateral hindpaws when PPT values were ≤80 g.

### 3.2. Animal Health

**Figure 2:**
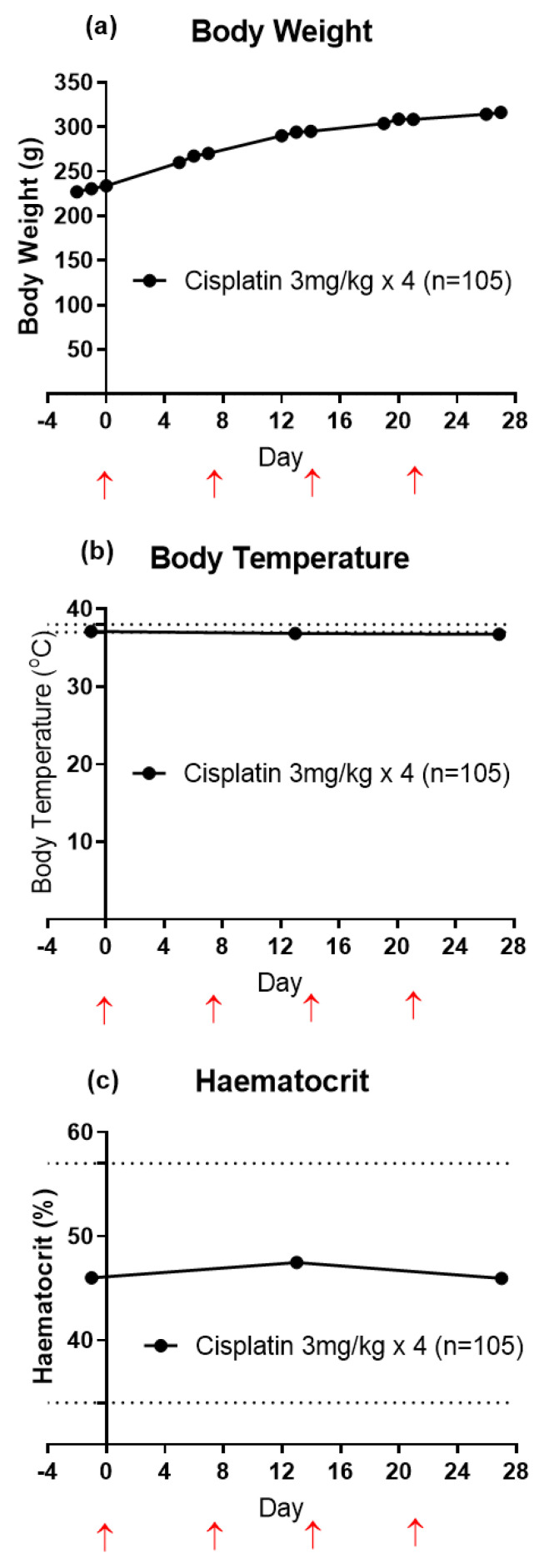
(**a**) Mean (±SEM) body weights of cisplatin-induced CIPN-rats commencing on day −2 and continuing for the 27 day study duration. (**b**) Mean (±SEM) body temperature of cisplatin-treated Sprague-Dawley rats from day −1 to 27. The normal body temperature range for rats (horizontally dotted lines) is 37 to 38 °C. (**c**) Mean (±SEM) blood haematocrit levels in cisplatin-induced CIPN-rats from day −1 to day 27. The normal haematocrit range for rats (horizontally dotted lines) is 34% to 57%. Red upward pointing arrows indicate the days of each cisplatin (3 mg/kg; IP) injection. Please note that the error bars have been added, but they cannot be seen as the data is very tight (n = 105).

The steady increase in the mean (±SEM) body weights of rats determined over the 27 day study period with cisplatin injections on days 0, 7, 14 and 21 indicates that the animals had good general health for the study duration (Figure 2a). For CIPN-rats, the mean (±SEM) body temperature values (n = 105) remained within the normal range (37 to 38 °C) [34] for the study duration (Figure 2b). Mean (±SEM) blood haematocrit levels of CIPN-rats were within the normal range between 34% to 57% [34] (Figure 2c). The urinalysis results are summarized in Table S1 (supplementary information) and these data further attest to the good general health status of the CIPN-animals.

### 3.3. Pharmacological Assessments

**Figure 3:**
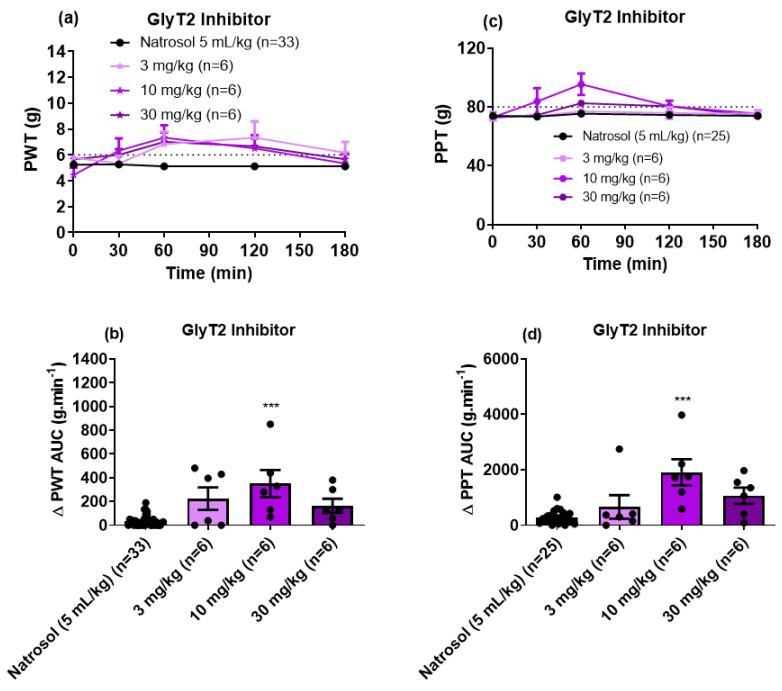
(**a**) Mean (±SEM) paw withdrawal threshold (PWT) versus time curves. (**b**) Scatter diagrams of the mean (±SEM) extent and duration of action quantified as the mean (±SEM) areas under the Δ PWT versus time curves (Δ PWT AUC values) for the combined left and right hindpaws of individual CIPN-rats following the administration of single PO bolus doses of the GlyT2 Inhibitor at 3 mg/kg (n = 6), 10 mg/kg (n = 6) and 30 mg/kg (n = 6), relative to vehicle (n = 33). The horizontal dotted line in Panel (**a**) indicates fully developed mechanical allodynia in the bilateral hindpaws (PWT ≤ 6 g). (**c**) Mean (±SEM) paw pressure threshold (PPT) versus time curves. (**d**) Scatter diagrams of the mean (±SEM) extent and duration of action quantified as the mean (±SEM) areas under the Δ PPT versus time curves (Δ PPT AUC values) for the combined left and right hindpaws of individual CIPN-rats following the administration of single oral bolus doses of the GlyT2 Inhibitor at 3 mg/kg (n = 6), 10 mg/kg (n = 6) and 30 mg/kg (n = 6) relative to vehicle (n = 25). The horizontal dotted line in Panel (**c**) indicates fully developed mechanical hyperalgesia in the bilateral hindpaws (PPT ≤ 80 g). *** *p* < 0.001.

For CIPN-rats with fully developed mechanical hypersensitivity in the bilateral hindpaws, single oral (PO) bolus doses of the GlyT2 inhibitor induced a bell-shaped dose–response curve for the relief of both mechanical allodynia and mechanical hyperalgesia (Figure 3a,b). It was only at the 10 mg/kg dose that the GlyT2 inhibitor significantly alleviated these pain-like behaviors. The corresponding mean durations of action were <3 h for mechanical allodynia and <2 h for mechanical hyperalgesia. The peak effect for both pain behaviors was at 1 h post-dose. An ED_50_ for the GlyT2 inhibitor was not attainable due to the bell-shaped dose–response curve. Consistent with expectations, single doses of vehicle (5 mL/kg; 0.5% Natrosol™ hydroxyethylcellulose and 0.01% Tween 80) lacked efficacy.

**Figure 4:**
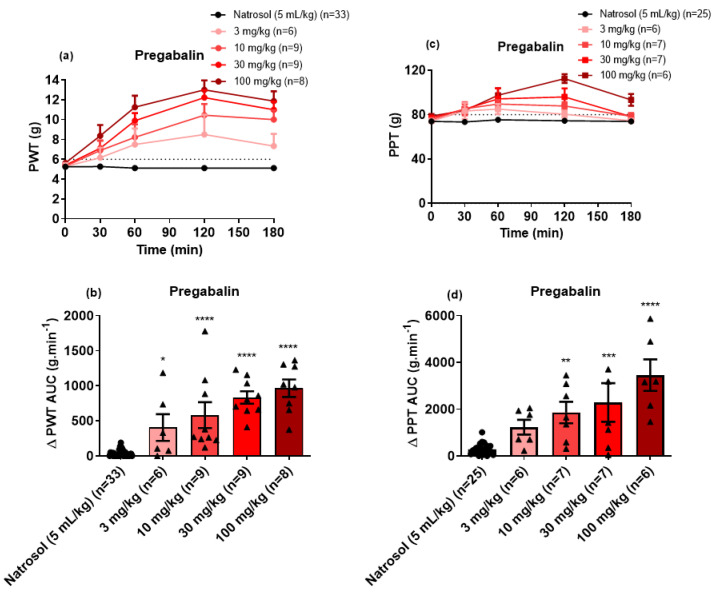
(**a**) Mean (±SEM) paw withdrawal threshold (PWT) versus time curves. (**b**) Scatter diagrams of the mean (±SEM) extent and duration of action quantified as the mean (±SEM) areas under the Δ PWT versus time curves (Δ PWT AUC values) for the combined left and right hindpaws of individual CIPN-rats following the administration of single oral bolus doses of pregabalin at 3 mg/kg (n = 6), 10 mg/kg (n = 9), 30 mg/kg (n = 9) and 100 mg/kg (n = 8) relative to vehicle (n = 33). The horizontal dotted line in Panel (**a**) indicates fully developed mechanical allodynia (PWT ≤ 6 g) in the bilateral hindpaws. (**c**) Mean (±SEM) paw pressure threshold (PPT) versus time curves. (**d**) Scatter diagrams of the mean (±SEM) extent and duration of action quantified as the mean (±SEM) areas under the Δ PPT versus time curves (Δ PPT AUC values) for the combined left and right hindpaws of individual CIPN-rats following the administration of single oral bolus doses of pregabalin at 3 mg/kg (n = 6), 10 mg/kg (n = 7), 30 mg/kg (n = 7) and 100 mg/kg (n = 6) relative to vehicle (n = 25). The horizontal dotted line in Panel (**c**) indicates fully developed mechanical hyperalgesia (PPT ≤ 80 g) in the bilateral hindpaws. * *p* ≤ 0.05, ** *p* < 0.01 ***, *p* < 0.001 and **** *p* < 0.0001.

By contrast with the bell-shaped dose–response curve for the GlyT2 inhibitor, single PO bolus doses of pregabalin (3 mg/kg to 100 mg/kg) induced dose-dependent relief of both mechanical allodynia (Figure 4a,b) and mechanical hyperalgesia (Figure 4c,d). The estimated ED_50_ (±95% CI) values for the relief of mechanical allodynia and mechanical hyperalgesia were 6.7 mg/kg (1.68 to 13.93) and 38.5 mg/kg (15.85 to 232.2), respectively. The peak anti-allodynic effect was observed at 2 h post-dose, while the peak anti-hyperalgesic effect was in the range 1 to 2 h. The mean durations of action for relief of mechanical allodynia and mechanical hyperalgesia were ≥3 h and ≤3 h, respectively.

**Figure 5:**
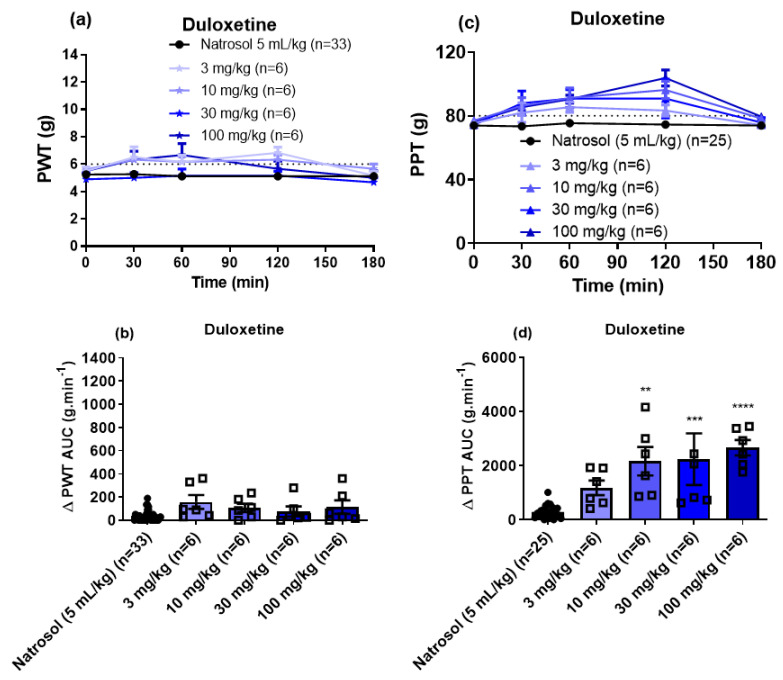
(**a**) Mean (±SEM) paw withdrawal threshold (PWT) versus time curves. (**b**) Scatter diagrams of the mean (±SEM) extent and duration of action quantified as the mean (±SEM) areas under the Δ PWT versus time curves (Δ PWT AUC values) for the combined left and right hindpaws of individual CIPN-rats following the administration of single oral bolus doses of duloxetine at 3 mg/kg (n = 6), 10 mg/kg (n = 6), 30 mg/kg (n = 6) and 100 mg/kg (n = 6) relative to vehicle (n = 33). The horizontal dotted line in Panel (**a**) indicates fully developed mechanical allodynia (PWT ≤ 6 g) in the bilateral hindpaws. (**c**) Mean (±SEM) paw pressure threshold (PPT) versus time curves. (**d**) Scatter diagrams of the mean (±SEM) extent and duration of action quantified as the mean (±SEM) areas under the Δ PPT versus time curves (Δ PPT AUC values) for the combined left and right hindpaws of individual CIPN-rats following the administration of single oral bolus doses of duloxetine at 3 mg/kg (n = 6), 10 mg/kg (n = 6), 30 mg/kg (n = 6) and 100 mg/kg (n = 6) at time 0 (pre-dosing) relative to vehicle (n = 25). The horizontal dotted line in Panel (**c**) indicates fully developed mechanical hyperalgesia (PPT ≤ 80 g) in the bilateral hindpaws. ** *p* < 0.01, *** *p* < 0.001 and **** *p* < 0.0001.

For the noradrenaline-serotonin reuptake inhibitor duloxetine, doses up to 100 mg/kg lacked efficacy for the relief of mechanical allodynia in the bilateral hindpaws of CIPN-rats (Figure 5a,b). However, duloxetine induced dose-dependent (10 to 100 mg/kg) relief of mechanical hyperalgesia in the bilateral hindpaws (Figure 5c,d). The peak anti-hyperalgesic effect was observed at 2 h post-dose and the corresponding duration of action was <3 h. The estimated ED_50_ (±95% CI) for the anti-hyperalgesic efficacy of duloxetine was 18.2 mg/kg (95% CI: 5.7 to 59.8).

**Figure 6:**
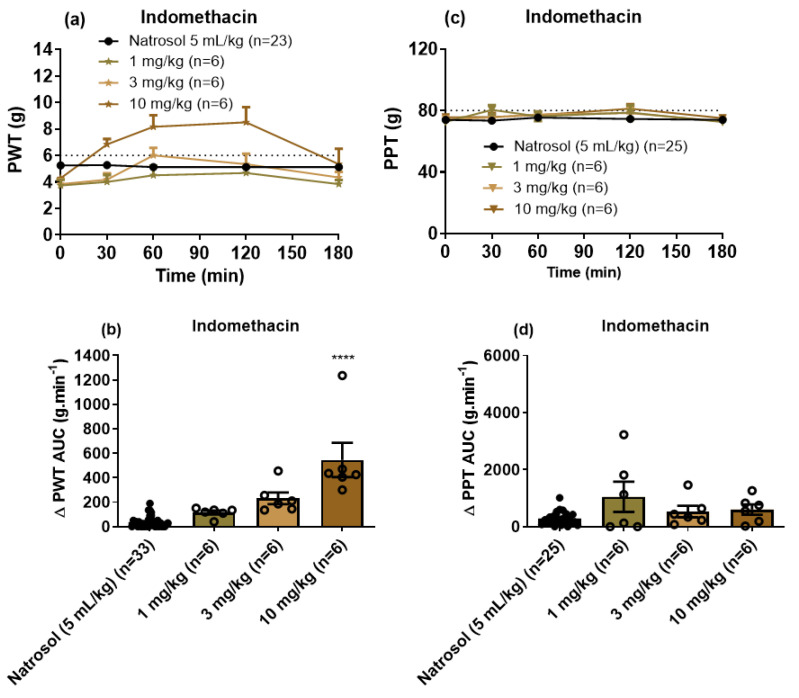
(**a**) Mean (±SEM) paw withdrawal threshold (PWT) versus time curves. (**b**) Scatter diagrams of the mean (±SEM) extent and duration of action quantified as the mean (±SEM) areas under the Δ PWT versus time curves (Δ PWT AUC values) for the combined left and right hindpaws of individual CIPN-rats following the administration of single oral bolus doses of indomethacin at 1 mg/kg (n = 6), 3 mg/kg (n = 6) and 10 mg/kg (n = 6) relative to vehicle (n = 33). The horizontal dotted line in Panel (**a**) indicates fully developed mechanical allodynia (PWT ≤ 6 g). (**c**) Mean (±SEM) paw pressure threshold (PPT) versus time curves. (**d**) Scatter diagrams of the mean (±SEM) extent and duration of action quantified as the mean (±SEM) areas under the Δ PPT versus time curves (Δ PPT AUC values) for the combined left and right hindpaws of individual CIPN-rats following the administration of single oral bolus doses of indomethacin at 1 mg/kg (n = 6), 3 mg/kg (n = 6) and 10 mg/kg (n = 6) relative to vehicle (n = 25). The horizontal dotted line in Panel (**c**) indicates fully developed mechanical hyperalgesia (PPT ≤ 80 g). **** *p* < 0.0001.

For indomethacin, a single bolus dose at 10 mg/kg induced significant relief of mechanical allodynia in the bilateral hindpaws of CIPN-rats (Figure 6a,b) with the peak effect observed at 1–2 h and a mean duration of action <3 h. However, indomethacin (1 to 10 mg/kg) lacked efficacy for the relief of mechanical hyperalgesia in the bilateral hindpaws (Figure 6c,d). The ED_50_ (±95% CI) for indomethacin for the relief of mechanical allodynia was not able to be determined.

The statistical analyses for the pain behavioral experiments are summarized in Table S2 (Supplementary information).

## 4. Discussion

In this investigation, we show for the first time that single PO bolus doses of the GlyT2 inhibitor of interest (Figure S1) induced a bell-shaped dose–response curve for the alleviation of mechanical allodynia and mechanical hyperalgesia in the bilateral hindpaws of CIPN-rats. Specifically, PO administration of the GlyT2 inhibitor at 10 mg/kg induced significant relief of both mechanical allodynia and mechanical hyperalgesia in the bilateral hindpaws while doses at 3 mg/kg and 30 mg/kg lacked efficacy (Figure 3). Our findings are reminiscent of the bell-shaped dose–response curve induced by intravenous bolus doses of the GlyT1 inhibitor ORG25935 for the relief of mechanical allodynia in the bilateral hindpaws of a mouse model of painful diabetic neuropathy (PDN). Specifically, ORG25935 was efficacious at a dose of 0.1 mg/kg, while both a lower dose (0.01 mg/kg) and larger doses (up to 10 mg/kg) lacked efficacy [37]. In unrelated Phase II clinical trials, bitopertin (another GlyT1 inhibitor) showed promising results on negative symptoms of schizophrenia at intermediate doses [38], with a bell-shaped dose–response curve for the bitopertin effects [39]. However, two Phase III clinical trials on the efficacy and safety of bitopertin for treating negative symptoms of schizophrenia failed to demonstrate a benefit of the drug over the placebo [40].

By contrast, the GlyT2 inhibitor ORG25543 induced dose-dependent anti-allodynia for the same dose range in the STZ-diabetic mouse of model of PDN as well as in a mouse model of partial sciatic nerve ligation induced neuropathic pain [37]. Our data herein differ from our previous findings in a rat model of prostate cancer-induced bone pain (PCIBP), whereby the same GlyT2 inhibitor used herein, induced dose-dependent relief of mechanical allodynia at single oral doses up to 30 mg/kg [27].

**Figure 7:**
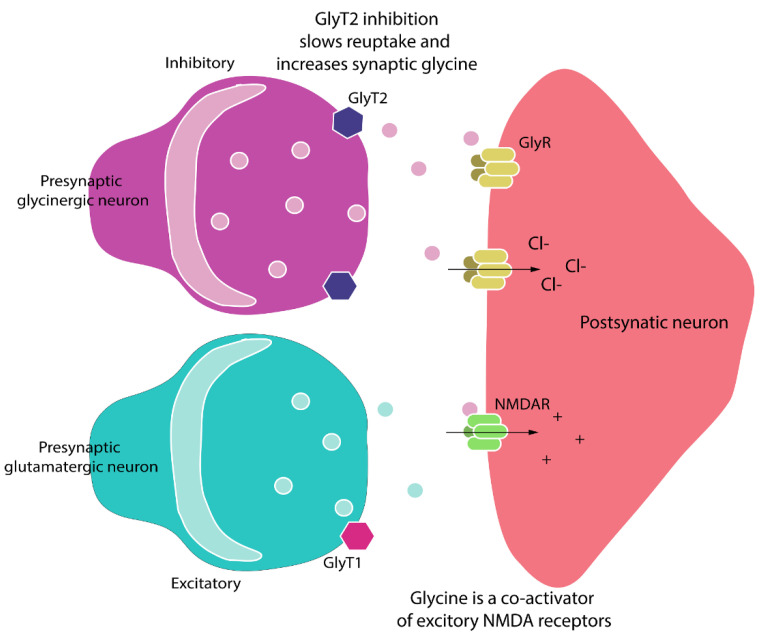
Pro-nociceptive signaling can be reduced by augmenting glycinergic activity in the spinal dorsal horn via inhibition of GlyT2 to increase synaptic glycine levels and increase inhibitory signaling through spinal glycine receptors (GlyRs). As glycine is a co-agonist for excitatory NMDA receptors (NMDARs), there is the potential for a “spill-over” pro-algesic effect through this latter mechanism [42].

These between-model differences in anti-allodynic efficacy of GlyT2 inhibitors are potentially due to the differences in the pathobiological mechanisms that underpin each of these persistent pain conditions. For example, PCIBP has both inflammatory and neuropathic components, whereas CIPN is a neuropathic pain condition. Other factors include differential impairment of inhibitory glycinergic transmission among the various persistent pain models. For example, in rodent models of inflammatory pain, the pro-inflammatory mediator PGE2 induced phosphorylation of the strychnine-sensitive glycine receptor α3 subunit in the superficial spinal dorsal horn and a PGE2-mediated reduction in glycinergic neurotransmission [28,41]. In other investigations, the timing of dose administration with respect to induction of the peripheral nerve injury had a marked effect on the extent to which anti-allodynia was induced by the GlyT2 inhibitors, ORG25543 and ALX1393 [37]. Exposure of mouse spinal cord slices to ORG25543 for >10 min resulted in a long-term reduction in inhibitory postsynaptic currents that is likely due to the blockade of glycine recycling. Furthermore, glycinergic currents were not restored after the washout of ORG25543 from oocytes expressing GlyT2, which is suggestive of the sustained complete block of GlyT2 to mimic the GlyT2 knockout mouse phenotype [37]. Another factor potentially contributing to the bell-shaped dose–response curve observed in CIPN-rats may be due to a “spill-over” pro-algesic effect at the 30 mg/kg dose as glycine is a co-agonist on post-synaptic NMDA receptors in the spinal cord and their activation will induce neuro-excitatory transmission rather than inhibitory transmission (Figure 7) [42].

In the present work, we found that our GlyT2 inhibitor tool compound at 10 mg/kg alleviated both mechanical allodynia and mechanical hyperalgesia in the bilateral hindpaws. Mechanical allodynia is a hallmark symptom of neuropathic pain, defined as pain induced by normally innocuous mechanical, thermal or proprioceptive stimuli [43]. In neuropathic pain states such as CIPN, activity of inhibitory neurons in the deep dorsal horn (lamina III and deeper) that would normally prevent pain due to Aβ-fiber stimulation in response to innocuous stimuli, such as light pressure or touch, appear to be compromised [44]. This is in line with work conducted by others whereby synaptic inhibition in the spinal dorsal horn was reduced in rodent models of neuropathic and chronic inflammatory pain [45,46]. This reduction in inhibition appears to be underpinned by reductions in both GABAergic and glycinergic mechanisms [47]. Thus, restoring inhibitory neurotransmission in the spinal dorsal horn by the inhibition of GlyT2 to augment glycinergic inhibitory signaling is expected to alleviate mechanical allodynia as was observed in CIPN-rats [47]. By contrast, mechanical hyperalgesia is an exaggerated painful sensation induced by noxious stimuli that are propagated by primary afferent Aδ and C fibers that synapse in superficial laminae I and II_o_ as well as laminae V and VI of the spinal cord [48]. Glycinergic neurons located in lamina I receive sensory input primarily from un-myelinated high threshold (nociceptive) sensory nerve fibers [47]. Lamina II contains only a few glycinergic neurons but, they nevertheless, express glycine receptors at high density [14] and also receive prominent glycinergic input [47]. Thus, based upon the anatomical location of glycinergic neurons in the spinal cord, we would expect GlyT2 inhibition to increase synaptic glycine levels and augment inhibitory glycinergic signaling in the spinal dorsal horn to alleviate both mechanical allodynia and mechanical hyperalgesia, as was observed in this study and in our previous study in a rat model of PCIBP [27]. Interestingly, recent work showed that combined subcutaneous administration of irreversible inhibitors of GlyT1 (NFPS at 1 mg/kg) and GlyT2 (ORG25543 at 2 mg/kg) induced an anti-allodynic effect in the partial sciatic nerve ligation rat model of neuropathic pain in contrast to the lack of efficacy of either GlyT inhibitors applied alone [32]. Thus, further investigation of dual GlyT1/GlyT2 inhibitors is warranted.

For the clinically used reference drugs administered to CIPN-rats herein, we assessed the anti-neuropathic efficacy of pregabalin, duloxetine and indomethacin that are representative compounds of three different analgesic/adjuvant drug classes [19]. Briefly, single oral bolus doses of pregabalin induced dose-dependent relief of both mechanical allodynia and mechanical hyperalgesia in the range 3–100 mg/kg. These findings are consistent with the use of pregabalin in the clinical setting for the relief of neuropathic pain [19]. By contrast, single oral bolus doses of the serotonin norepinephrine reuptake inhibitor, duloxetine (3–100 mg/kg) lacked efficacy for the relief of mechanical allodynia in the bilateral hindpaws of our rat model of CIPN, whereas it was efficacious for the relief of mechanical hyperalgesia in the bilateral hindpaws of this model. These findings are consistent with the moderate recommendation for duloxetine in the ASCO guideline for the treatment of established CIPN [22].

Although single oral bolus doses of the non-steroidal anti-inflammatory drug (NSAID) indomethacin at 10 mg/kg alleviated mechanical allodynia in the bilateral hindpaws of CIPN-rats, it lacked efficacy for the relief of mechanical hyperalgesia in the bilateral hindpaws of these animals. This latter effect is aligned with clinical experience showing that NSAIDs do not alleviate neuropathic pain in patients [49,50].

In CIPN-rats used in this present study, there were no discernible side effects induced by our GlyT2 inhibitor, which is in agreement with our previous observations in a rat model of PCIBP [27]. In a safety study, it was found that the irreversible GlyT2 inhibitor ORG25543 had an overall excitatory profile that is characterized by a dose-dependent increase in the incidence of neuroexcitatory side effects including tremors and stereotypies [31]. By comparing the safety profiles of ORG25543 to the reversible GlyT2 inhibitor ALX1393, it was proposed that reversible GlyT inhibitors may allow a tolerable balance between efficacy and toxicity [31].

It has been proposed that enhanced glycinergic signaling in the spinal dorsal horn may have substantial advantages compared to augmented GABAergic signaling, due to its profound caudo-rostral gradient with strong innervation of the spinal cord and hindbrain and weaker innervation of the midbrain and forebrain [51]. Additionally, approximately 75% of the inhibitory input onto excitatory neurons of the superficial dorsal horn is glycinergic and only 25% is GABAergic [52]. Finally, since glycine and GABA are often co-released from the same presynaptic terminals, potentiation of glycine receptor function should restore inhibition even if the inhibitory loss was predominantly GABAergic [47].

Overall, CIPN-rats in this investigation had good general health. This is indicated by the increase in the mean (±) body weight over the 4 week cisplatin-dosing period and the maintenance of mean (±SEM) body temperature and haematocrit levels within the normal range. In our study, we hyper-hydrated rats with a 2 mL s.c. saline injection at 5 min prior to each cisplatin injection to prevent renal damage [34,35]. Our success in this regard is attested by the urinalysis data shown in Table S1 (Supplementary Information).

In conclusion, the bell-shaped dose–response curve of the orally active GlyT2 inhibitor tool compound assessed in this investigation in CIPN-rats suggests that future work directed at development of mixed GlyT1/GlyT2 inhibitors may be fruitful. This is because GlyT1 inhibition has the potential to reduce the “spill-over” pro-algesic effect of GlyT2 inhibition that is underpinned by increased glutamatergic neurotransmission transduced through NMDA receptors. This notion is supported by the recent work of others showing that combined subcutaneous dosing with irreversible inhibitors of GlyT1 and GlyT2 induced anti-allodynia effects in a rat model of neuropathic pain that is in contrast to either GlyT inhibitor alone [32].

## Data Availability

The data are available from the corresponding author upon request.

## Supplementary Materials

The following are available online at https://www.mdpi.com/article/10.3390/biom11070940/s1, Figure S1: Chemical structure of the GlyT2 inhibitor, Table S1: Effects of the cisplatin dosing regimen (4 i.p. doses at 3 mg/kg at once-weekly intervals) on urine indices of kidney function in male SD rats over a 4 week study period, Table S2: Summary of the statistical analysis of the efficacy data for single oral bolus does of the GlyT2 inhibitor relative to pregabalin, duloxetine, indomethacin and vehicle for alleviating mechanical allodynia and mechanical hyperalgesia in the bilateral hindpaws of CIPN-rats.
