# Supplementary material for: Assessment of the Anti-Allodynic and Anti-Hyperalgesic Efficacy of a Glycine Transporter 2 Inhibitor Relative to Pregabalin, Duloxetine and Indomethacin in a Rat Model of Cisplatin-Induced Peripheral Neuropathy
Source: Biomolecules. 2021 Jun 24;11(7):940. doi: 10.3390/biom11070940 (PMC8301897; doi:10.3390/biom11070940)
Supplement: Supplementary file 1 [file biomolecules-11-00940-s001.zip › biomolecules-1246499-supplementary.pdf]

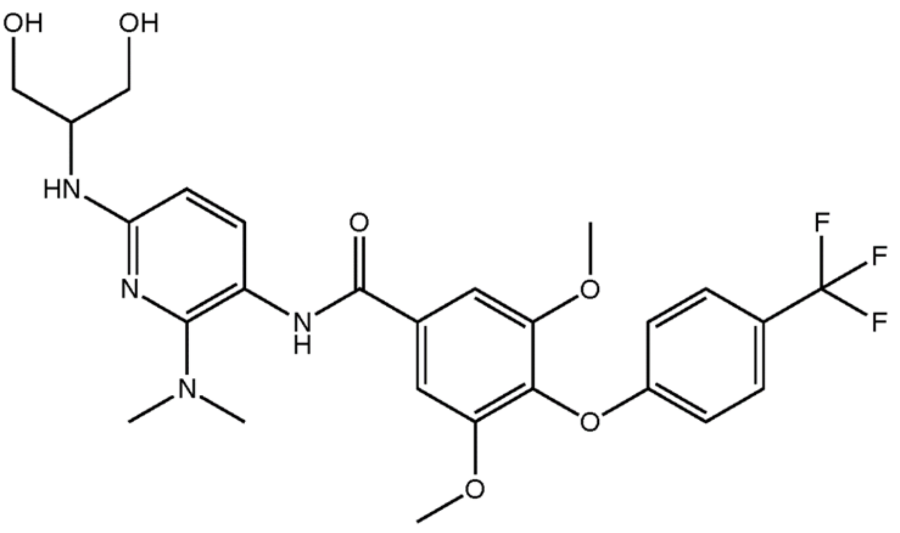

N-(6-((1,3-dihydroxypropan-2-yl)amino)-2-(dimethylamino)pyridin-3-yl)-3,5-dimethoxy-4-(4-(trifluoromethyl)phenoxy)benzamide

Figure S1. Chemical structure of the GlyT2 inhibitor.

Table S1. Effects of the cisplatin dosing regimen (4 i.p. doses at 3 mg/kg at once-weekly intervals) on urine indices of kidney function in male SD rats over a 4-week study period.

**Day Post-Cisplatin (n=105) treatment**	**-2**	**12**	**26**
**Glucose<br>(mg/dL, mmol/L)**	-ve	-ve (n=90)<br>Trace 100, 5.5 (n=5)<br>+ 250, 14 (n=2)<br>++ 500, 28 (n=5)<br>+++ 1000, 55(n=3)	-ve (n=104)<br>Trace 100, 5.5 (n=1)
**Bilirubin**	-ve	-ve	-ve (n=104)<br>+ Small (n=1)
**Ketone<br>(mg/dL, mmol/L)**	-ve (n=104)<br>Small 15, 1.5 (n=1)	-ve (n=94)<br>Trace 5, 0.5 (n=9)<br>Small 15, 1.5 (n=2)	-ve (n=97)<br>Trace 5, 0.5 (n=7)<br>Small 15, 1.5 (n=1)
**Specific Gravity<br>(Mean ± SEM)**	1.005 ± 0.000	1.008 ± 0.000	1.008 ± 0.000
**Erythrocytes<br>(ca Cells/uL)**	-ve (n=95)<br>Trace 10 (n=4)*<br>Trace (n=6)^	-ve (n=97)<br>Trace 10 (n=5)*<br>Small 25 (n=2)^<br>Large 200 (n=1)^	-ve (n=97)<br>Trace 10 (n=3)*<br>Moderate 80 (n=2)*<br>Trace (n=2)^<br>Large 200 (n=1)^
**pH<br>(Mean ± SEM)**	8.15 ± 0.04	7.79 ± 0.059	7.53 ± 0.069
**Protein<br>(mg/dL, g/L)**	-ve (n=58)<br>Trace (n=36)<br>+ 30, 0.3 (n=9)<br>++ 100, 1 (n=2)	-ve (n=13)<br>Trace (n=32)<br>+ 30, 0.3 (n=49)<br>++ 100, 1 (n=10)<br>+++ 300, 3 (n=1)	-ve (n=18)<br>Trace (n=45)<br>+ 30, 0.3 (n=40)<br>++ 100, 1 (n=2)
**Urobilinogen<br>(Ehrlich units/dL urine, umol/L)**	Normal 0.2, 3.2	Normal 0.2, 3.2	Normal 0.2, 3.2

**Nitrite**	-ve (n=102)<br>+ve (n=3)	-ve	-ve (n=99)<br>+ve (n=6)
**Leukocytes<br>(ca Cells/uL)**	-ve (n=91)<br>Trace 15 (n=14)	-ve (n=5)<br>Trace 15 (n=42)<br>+ 70 (n=37)<br>++ 125 (n=20)<br>+++ 500 (n=1)	-ve (n=2)<br>Trace 15 (n=40)<br>+ 70 (n=43)<br>++ 125 (n=19)<br>+++ 500 (n=1)

* non-haemolysed; ^ haemolysed.

For the urinalysis, low to high values of each parameter are defined as follows:

Glucose: (1) Neg (negative); (2) 100 mg/dL, 5.5 mmol/L, Trace; (3) 250 mg/dL, 14 mmol/L, +; (4) 500 mg/dL, 28 mmol/L, ++; (5) 1000 mg/dL, 55 mmol/L, +++ and (6) ⩾2000 mg/dL, ⩾111 mmol/L, ++++.

Bilirubin: (1) Neg; (2) Small, +; (3) Moderate, ++ and (4) Large, ++++.

Ketone: (1) Neg; (2) Trace, 5 mg/dL, 0.5 mmol/L; (3) Small, 15 mg/dL, 1.5 mmol/L; (4) Moderate, 40 mg/dL, 4 mmol/L; (5) Large, 80 mg/dL, 8 mmol/L and (6) Large, 160 mg/dL, 16 mmol/L.

Blood (erythrocytes): (1) Neg; (2) Trace, 10 ca Cells/μL (non-haemolysed; orange colour); (3) moderate, 80 ca Cells/μL (non-haemolysed; orange colour); (4) Trace (haemolysed, light green colour); (5) small, 25 ca Cells/μL (haemolysed, light green colour); (6) moderate, 80 ca Cells/μL (haemolysed, green colour) and (7) Large, 200 ca Cells/μL (haemolysed, dark green colour).

Specific Gravity: (1) 1.000; (2) 1.005; (3) 1.010; (4) 1.015; (5) 1.020; (6) 1.025 and (7) 1.030.

pH: (1) 5.0; (2) 6.0; (3) 6.5; (4) 7.0; (5) 7.5; (6) 8.0 and (7) 8.5.

Protein: (1) Neg; (2) Trace; (3) 30 mg/dL, 0.30 g/L, +; (4) 100 mg/dL, 1 g/L, ++; (5) 300 mg/dL, 3 g/L, +++ and (6) ⩾200 mg/dL, ⩾20 g/L, ++++.

Urobilinogen: (1) 0.2 Ehrlich Units/dL Urine, 3.2 μmol/L, Normal; (2) 1, 16, Normal; (3) 2, 33; (4) 4, 66 and (5) 8, 131.

Nitrite: (1) Neg and (2) Positive (any degree of uniform pink colour).

Leucocytes: (1) Neg; (2) 15 ca Cells/μL, Trace; (3) 70 ca Cells/μL, +; (4) 125 ca Cells/μL, ++ and (5) 500 ca Cells/μL, +++.

Table S2. Summary of the efficacy of single oral bolus does of the GlyT2 inhibitor relative to pregabalin, duloxetine, indomethacin and vehicle for alleviating mechanical allodynia and mechanical hyperalgesia in the bilateral hindpaws of CIPN-rats.

	**Mechnical Allodynia**	**Mechnical Hyperalgesia**
**0.5% Natrosol/0.01% Tween 80**		
**5 mL/kg**	NA	NA
**A novel GlyT2 inhibitor**		
3 mg/kg	NS	NS
10 mg/kg	***	***
30 mg/kg	NS	NS
**Pregabalin**		
3 mg/kg	*	NS
10 mg/kg	****	**
30 mg/kg	****	***
100 mg/kg	****	****
**Duloxetine**		
3 mg/kg	NS	NS
10 mg/kg	NS	**
30 mg/kg	NS	***
100 mg/kg	NS	****
**Indomethacin**		
1 mg/kg	NS	NS
3 mg/kg	NS	NS
10 mg/kg	****	NS

NA not applicable; NS not significant; *$p \leqslant 0.05$ ** $p < 0.01$ *** $p < 0.001$ ****$p < 0.0001$.
